# APOBEC3B protein expression associates with poor prognosis for breast cancer patients with ER-positive disease

**DOI:** 10.1186/s13058-025-02167-7

**Published:** 2025-12-09

**Authors:** Maartje A. C. Schreurs, A. Mieke Timmermans, Michael A. Carpenter, Marcel Smid, Michael A. den Bakker, Carolien H. M. van Deurzen, Reuben S. Harris, John W. M. Martens

**Affiliations:** 1 Department of Medical Oncology, Erasmus MC Cancer Institute, Erasmus University Medical Center, Rotterdam, The Netherlands; 2 Department of Biochemistry and Structural Biology, University of Texas Health San Antonio, San Antonio, Texas 78229 USA; 3 Howard Hughes Medical Institute, University of Texas Health San Antonio, San Antonio, Texas 78229 USA; 4 Department of Pathology, Maasstad Hospital, Rotterdam, The Netherlands; 5 Department of Pathology, Erasmus MC Cancer Institute, Erasmus University Medical Centre, Rotterdam, The Netherlands

## Abstract

APOBEC enzymes, including APOBEC3B (A3B), have been linked to recurrent mutational patterns in breast cancers (BC). Currently, there is considerable level of evidence that A3B mRNA expression is associated with poor prognosis in ER-positive BC. Since the clinical relevance of A3B at the protein level has not been well studied, we evaluated A3B protein expression by immunohistochemistry on tumor tissue microarrays. Samples from 646 invasive, non-metastatic primary ER-positive BC patients who did not receive any systemic endocrine therapy as standard of care treatment, allowed us to study the pure prognostic impact of A3B protein expression on disease-free survival (DFS), metastatic disease-free survival (MFS), breast cancer specific-survival (BCSS), and overall survival (OS). Furthermore, 220 tumor specimens from BC patients who relapsed and did receive tamoxifen as first-line palliative treatment were included to study the association of A3B protein expression and progression free survival (PFS). Cox regression models were used to determine the impact of A3B protein expression on prognosis and PFS. We found that A3B expression associates with larger and higher grade tumors. Furthermore, univariable analyses showed that high A3B expression was associates with a shorter DFS (HR = 1.68; 95%CI = 1.31–2.14), MFS (HR = 1.93; 95%CI = 1.45–2.56), BCSS (HR = 2.72; 95%CI = 1.89–3.90), and OS (HR = 1.89; 95%CI = 1.40–2.56). Also, a shorter PFS (HR = 1.41; 95%CI = 1.03–1.92) was found. After adjustment for confounders, the observed associations remained significant. To conclude, in ER-positive BC, A3B protein expression is a marker for poor disease outcome and for rapid progression during endocrine treatment.

**Supplementary Information:**

The online version contains supplementary material available at 10.1186/s13058-025-02167-7.

## Introduction

Breast cancer (BC) is the most frequent cancer in women worldwide, both in cases and in deaths [1]. It is a heterogenous disease where the molecular determinants of a tumor determine whether that tumor is aggressive and which treatment regimens are effective [2]. To further understand the different subgroups of breast tumors, genomic studies have been conducted [3]. As a result, patterns of somatic mutations also called ‘mutational signatures’ have been found [4, 5]. Single-base substitution (SBS) mutational signatures 2 and 13 are caused by Apolipoprotein B mRNA editing enzyme catalytic polypeptide-like (APOBEC) enzymes [6–9]. These enzymes normally serve an important role in the innate immune defense against a wide variety of viral pathogens and transposons [6, 7, 10, 11].

APOBEC signatures have been observed in 15% of the primary BC and 25% of the recurrent diseases [6, 12, 13]. Our genome encodes seven distinct APOBEC3 enzymes (A, B, C, D, F, G and H), with molecular evidence highlighting APOBEC3B (A3B) as a strong candidate for involvement in BC [8, 14]. Elevated levels of A3B mRNA expression are associated with increased levels of cell proliferation [15, 16]. As a result, BC patients more often experience undesirable outcomes, such as recurrences, metastatic disease and poor survival [17–19], especially in ER-positive BC [15, 18].

Furthermore, studies have shown that A3B is associated with therapy resistant tumors [14, 16–21]. One study in ER-positive BC patients showed that A3B mRNA expression was associated with endocrine treatment failure, suggesting that this is less suitable for A3B-positive BC [15]. Therefore, a recent review proposed A3B as a predictive biomarker in several BC research plans [22].

Due to the rapid advancement in RNA profiling technologies (real time PCR, gene expression arrays and RNA sequencing), the majority of studies have focused on A3B mRNA expression. However, due to the imperfect relation between mRNA expression and APOBEC mutagenesis, analysis of A3B protein expression is pivotal. Furthermore, in daily clinical practice immunohistochemical staining on formalin-fixed paraffin-embedded (FFPE) tissues is widely established and still an important tool in the detection of biomarkers.

In this study, we determined A3B protein expression on FFPE tissues by immunohistochemical staining of tumor microarrays (TMA) using the rabbit α-human A3B monoclonal antibody 5210–87-13 [23] allowing us to study the impact of A3B expression on prognosis and progression-free survival (PFS). Ultimately, we aim to substantiate at the protein level the reported predictive value of mRNA expression of A3B in FFPE tissues and determine whether the A3B protein expression corresponds with a poor prognosis in ER-positive BC patients.

## Methods

### Cohort

FFPE primary breast tumors tissues were collected from all patients with BC who entered the Erasmus University Medical Center (Rotterdam, the Netherlands) for their local treatment between 1985 and 2000 and for which the required follow-up were available. Our institution’s Medical Ethical Committee approved our protocol for studying molecular markers associated with disease recurrence in anonymized tumor tissues (MEC 02–953). Furthermore, in this study, we adhere to the Code of Conduct of the Federation of Medical Scientific Societies in the Netherlands (http://www.fmwv.nl/).

From 1340 patients FFPE tissues were stained and complete clinical follow-up was collected for the primary objective. For this study, 646 samples from invasive, non-metastatic, ER-positive BC patients were stained to determine the A3B expression. Of those, 384 tissues were from patients with lymph node negative disease and who did not receive (neo-)adjuvant treatment according to the guidelines in the Netherlands at that time, and hence could be used to assess the pure prognostic value of A3B protein expression. For the primary objective we analyzed the relation of A3B protein expression with disease-free survival (DFS), metastatic disease-free survival (MFS), BC-specific survival (BCSS) and overall survival (OS) as outcome measures.

Furthermore, to assess the role of A3B in response to the first-line tamoxifen treatment, an additional set of 220 primary tumor FFPE tissues of endocrine treatment naïve BC patients were collected. These patients had recurrent disease and A3B expression was related to progression-free survival (PFS) after first-line tamoxifen treatment (standard of care in the Netherlands before the year 2000). Both cohorts have been previously described in detail [24, 25].

### Tissue microarray and immunohistochemistry

Two pathologists (MAdB, CHMvD) assessed the histology of all eligible tumors and scored tumor grade according to the modified method of Scarff, Bloom & Richardson prior to preparing the tissue microarrays (TMA). Thereafter, representative areas of the tumor for inclusion in the TMA were marked. From those areas, three cores were taken with a 0.6mm needle and added to the TMA. TMAs were stained with standard protocols and as described in more details in previous study for ER, PR, HER2 [24].

TMAs were subjected to heat-based antigen retrieval for 40 min using a high pH buffer solution (DAKO, Agilent Technologies Inc, Santa Clara, CA, USA). After which, they were cooled down to room temperature in 20 min and washed with PBS buffer, blocking with antibody diluent background reducing (Agilent Technologies Inc, Santa Clara, CA, USA). Subsequently, they were incubated with the polyclonal rabbit antibody 5210–87-13 against human A3B (dilution 1:160) overnight [23]. This antibody recognizes a shared C-terminal epitope in APOBEC3A (A3A), A3B and APOBEC3G (A3G). Since A3A and A3G are rarely expressed in breast tumors at the protein level [23], this antibody is appropriate for the purpose of this study.

We used breast cell lines also present in TMA blocks as our controls for high positive (BT474, MM361), low positive and negative controls (UACC812 & DU4475, CAMA, SKBR7). Furthermore, normal tissues present in TMAs were used as a positive control (stomach, intestine) and negative control (brain, liver, kidney). Protein expression was blindly scored on at least two cores for quantity (0%, 1–10%, 11–25%, 25–50% and more than 50%) by AMT.

### Statistical analysis

Statistical analyses were performed using STATA software (version 18). A Chi-square test was performed to assess associations between the APOBEC3B protein expression categories and clinicopathological data. In addition, non-parametric tests for trend were performed to study the association between A3B protein expression classifications and clinical characteristics.

Cox regression models were used for the survival analyses. Kaplan–Meier curves were presented for visualization. Follow-up started at the time of BC surgery and ended at the occurrence of recurrent disease, metastatic disease or death, depending on the outcome of interest. Patients were censored at the last date of contact without any event present, with a maximum of 10 years since time of diagnosis. For MFS, all distant metastases were counted as an event, but local–regional relapses were not. Patients diagnosed with secondary contralateral BC were censored for MFS at the date of diagnosis of the secondary BC. For BCSS, we used the cause of death that was reported. If this was not available, and a patient was diagnosed with metastatic disease before death, this patient would be coded as BC-specific death. In addition, we performed a sensitivity analysis for all lymph node negative BC patients who did not receive any (neo) adjuvant treatment for their primary BC. This allowed us to study the pure impact of A3B protein expression on the prognosis. Univariable as well as multivariable Cox regression analyses adjusted for age at diagnosis, menopausal status, nodal status, tumor size, tumor grade, and chemotherapy were performed.

For the PFS, follow-up started at the start of first-line treatment and ended at the time of progression of disease or death, with a maximum of 36 months after start of treatment. Univariable as well as multivariable Cox regression analyses adjusted for disease-free interval and dominant site of relapse were performed.

## Results

### Tumor microarray scoring

**Fig. 1:**
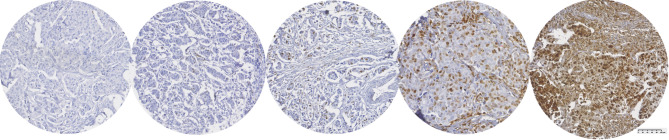
Immunohistochemical stained tissues showing the absence/presence of APOBEC3B protein. From left to right: negative (no expression), < 10%, 11–25%, 26–50% and > 50% protein expression

In Fig. 1, examples of the quantity scoring are provided. For this study, we dichotomized the A3B expression based quantity in ≤ 10% as A3B-low and > 10% as A3B-high. Additional analyses were conducted to further understand the increasing A3B protein expression by categorizing expression into ≤ 10% (A3B-L (low)); 11–25% (A3B-I (intermediate)) and > 25% (A3B-H (high)), which is presented in the supplementary material.

### Prognostic value of A3B protein expression in ER-positive BC

#### Patient characteristics

In total, 646 samples from ER-positive BC patients were eligible for the analysis on prognosis. The median age at diagnosis was 55 years (range 26–92). A total of 396 (61.3%) tumor samples were A3B-low and 250 (38.7%) were A3B-high. 230 out of the 396 A3B-low samples had no A3B expression. In the A3B-high group, 125 samples had expression in 11–25% of the cells, 64 in 26–50% of the cells, and 61 in more than 50% of the cells. Table 1 provides an overview of all clinical characteristics of this population. In this population, A3B-high breast tumors were more often diagnosed with a larger tumor size (50.4% vs. 29.6%), had more frequent higher tumor grade 2/3 (90.0% vs. 67.4%), and higher mitotic rates (11.9 ± 13.0 vs. 5.0 ± 7.4) than A3B-low tumors (all p-value < 0.001). When samples were further stratified as A3B-L (0–10%, n = 396), A3B-I (11–25%, n = 125) and A3B-H (> 25%, n = 125), similar results are found showing a trend where higher A3B protein expression was associated with larger tumors, higher tumor grade and higher rates of mitotic activity (Supplementary Table 1).

**Table 1:** Characteristics of the 646 ER-positive, non-metastatic breast cancer patients included in the analysis and the test for trend between the characteristics and APOBEC3B protein expression

	Low (n = 396)	High (n = 250)	Test for trend *p*-value
Age			0.52
Median (range)	55 (27–92)	55 (26–86)	
Mean ± SD	55.8 ± 12.4	55.2 ± 12.7	
Age category, n (%)			0.55
≤ 40	39 (9.9)	26 (10.4)	
41–55	165 (41.7)	109 (43.6)	
> 55	192 (48.5)	115 (46.0)	
Menopausal status, n (%)			0.77
Premenopausal	217 (54.8)	140 (56.0)	
Postmenopausal	179 (45.2)	110 (44.0)	
Tumor size, n (%)			< 0.001
pT1	279 (70.5)	124 (49.6)	
pT2/pT3	117 (29.6)	126 (50.4)	
Tumor grade, n (%)			< 0.001
1	129 (32.6)	25 (10.0)	
2/3	267 (67.4)	225 (90.0)	
Chemotherapy, n (%)			0.22
No or not applicable	317 (80.1)	190 (760)	
Yes	79 (20.0)	60 (24.0)	
Mitotic activity per mm^2^			< 0.001
Median (range)	3 (0–70)	8 (0–90)	
Mean ± SD	5.1 ± 7.4	11.9 ± 13.0	
Mitotic activity per mm^2^, n (%)			< 0.001
0–10	345 (87.6)	149 (59.6)	
> 10	49 (12.4)	101 (40.4)	

Low APOBEC3B protein expression is defined as 0–10%; high protein expression is defined as > 10%. Percentages may not add up to 100% due to rounding

#### Association with survival outcomes

**Fig. 2:**
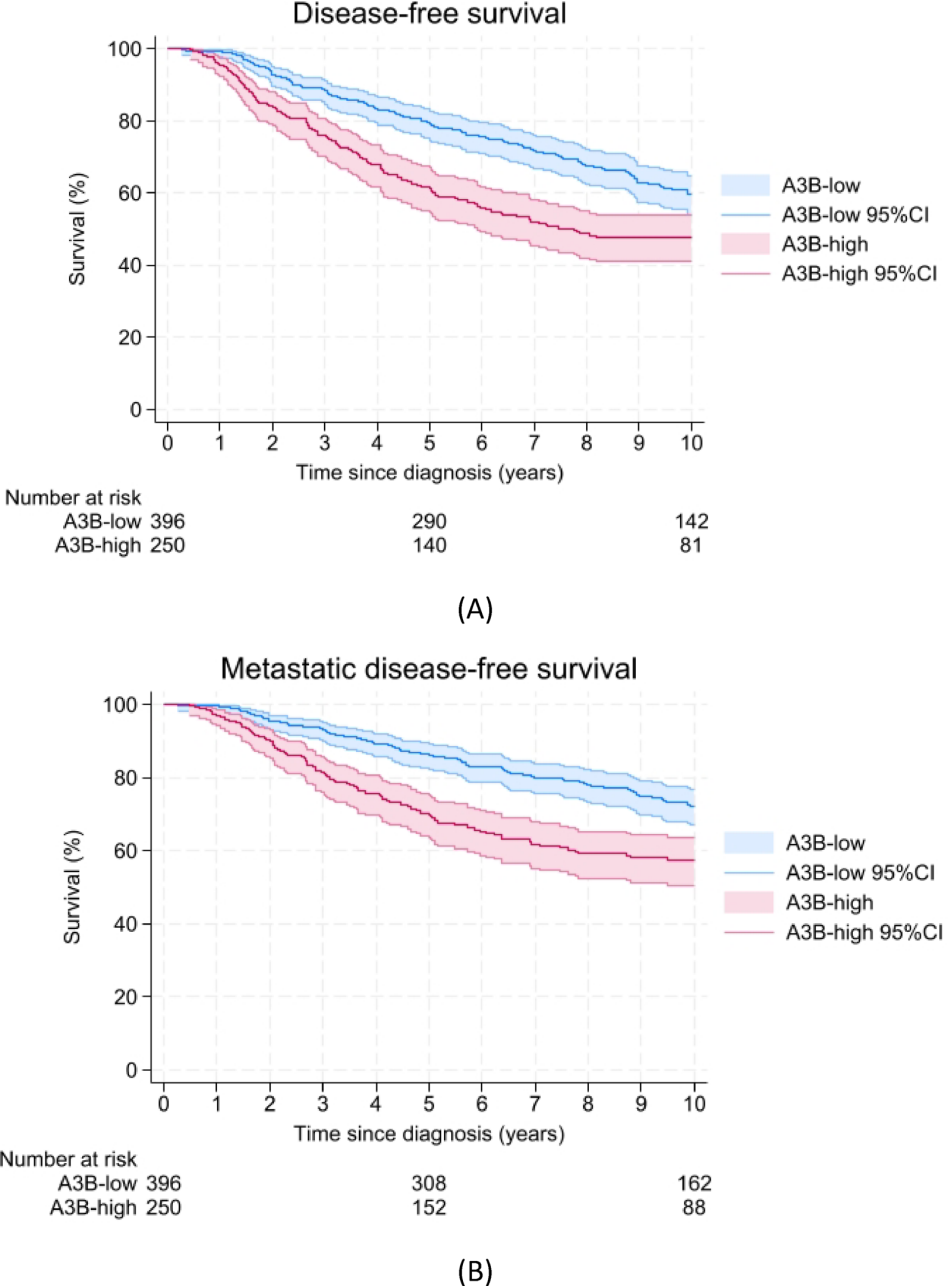

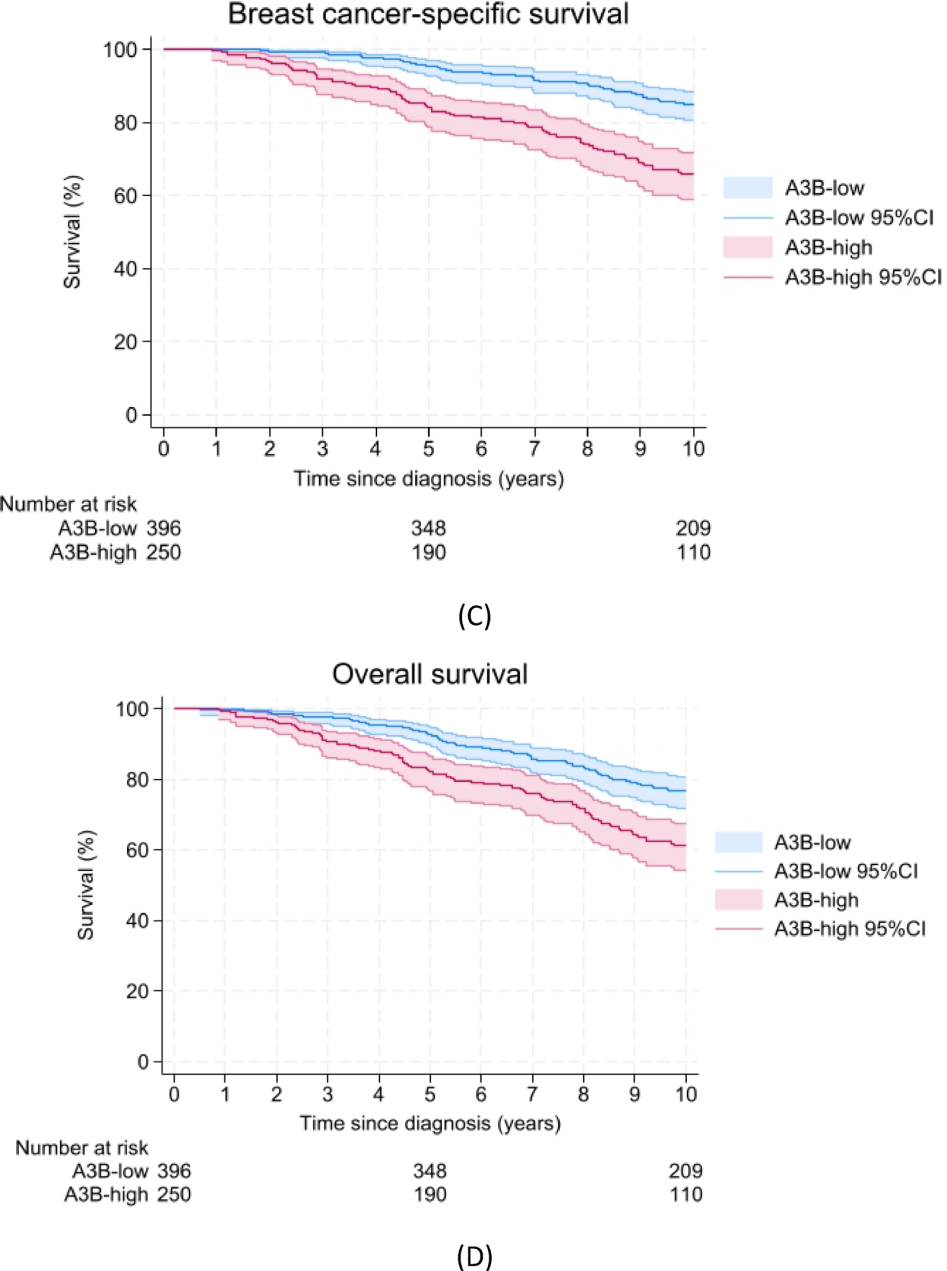
Survival curves representing **A** disease-free survival, **B** metastatic disease-free survival, **C** breast cancer-specific survival, and **D** overall survival up to 10 years after diagnosis with ER-positive breast cancer. 95%CI = 95% confidence interval

In the univariable analyses, we found that A3B-high samples were associated with a shorter DFS (HR = 1.68; 95%CI = 1.31–2.14, log-rank test *p* < 0.001, Fig. 2A), MFS (HR = 1.93; 95%CI = 1.45–2.56, log-rank test *p* < 0.001, Fig. 2B), BCSS (HR = 2.72; 95%CI = 1.89–3.90, log-rank test *p* < 0.001, Fig. 2C) and OS (HR = 1.89; 95%CI = 1.40–2.56, log-rank test *p* < 0.001, Fig. 2D) compared to the A3B-low samples. After adjustment for age at diagnosis, menopausal status, tumor size, tumor grade and chemotherapy as potential confounders in the multivariable analysis, A3B-high samples were again associated with a shorter DFS (HR = 1.37; 95%CI = 1.06–1.77), MFS (HR = 1.42; 95%CI = 1.05–1.91), BCSS (HR = 1.93; 95%CI = 1.33–2.81) and OS (HR = 1.45; 95%CI = 1.06–1.99; Table 2).

**Table 2:** Univariable and multivariable analysis for disease-free survival, metastatic-free survival, breast cancer-specific survival and overall survival in 645 ER-positive breast cancer patients

	Disease-free survival				Metastatic disease-free survival			
	Univariable analysis		Multivariable analysis		Univariable analysis		Multivariable analysis	
	HR (95%CI)	*P*-value	HR (95%CI)	*P*-value	HR (95%CI)	*P*-value	HR (95%CI)	*P*-value
Age, each year increase	0.99 (0.98–1.00)	< 0.01	0.98 (0.97–1.00)	0.09	0.98 (0.97–1.00)	0.01	0.98 (0.96–1.00)	0.11
*Menopausal status*								
Post- vs premenopausal	0.83 (0.65–1.06)	0.14	1.21 (0.81–1.83)	0.35	0.86 (0.64–1.14)	0.29	1.37 (0.84–2.22)	0.21
*Tumor size*								
pT2/pT3 vs. pT1	1.71 (1.34–2.18)	< 0.001	1.48 (1.15–1.90)	< 0.01	2.20 (1.66–2.93)	< 0.001	1.78 (1.32–2.40)	< 0.001
*Tumor grade (BR)*								
2/3 vs. 1	1.99 (1.43–2.76)	< 0.001	1.68 (1.19–2.35)	< 0.01	3.03 (1.92–4.77)	< 0.001	2.40 (1.51–3.83)	< 0.001
*Chemotherapy*								
Yes vs. no	1.64 (1.26–2.14)	< 0.001	1.41 (1.04–1.90)	0.03	2.00 (1.48–2.71)	< 0.001	1.70 (1.21–2.40)	< 0.01
*APOBEC3B expression*								
High vs. Low	1.68 (1.31–2.14)	< 0.001	1.37 (1.06–1.77)	0.02	1.93 (1.45–2.56)	< 0.001	1.42 (1.05–1.91)	0.02

	Breast cancer-specific survival				Overall survival			
	Univariable analysis		Multivariable analysis		Univariable analysis		Multivariable analysis	
	HR (95%CI)	*P*-value	HR (95%CI)	*P*-value	HR (95%CI)	*P*-value	HR (95%CI)	*P*-value
Age, each year increase	0.98 (0.96–0.99)	< 0.01	0.99 (0.97–1.02)	0.54	1.01 (0.99–1.02)	0.34	1.02 (1.00–1.04)	0.06
*Menopausal status*								
Post- vs premenopausal	0.67 (0.47–0.96)	0.03	0.92 (0.50–1.68)	0.79	1.10 (0.81–149)	0.54	0.88 (0.52–1.47)	0.62
*Tumor size*								
pT2/pT3 vs. pT1	2.98 (2.07–4.28)	< 0.001	2.17 (1.49–3.16)	< 0.001	2.36 (1.74–3.20)	< 0.001	1.87 (1.36–2.56)	< 0.001
*Tumor grade (BR)*								
2/3 vs. 1	4.69 (2.38–9.24)	< 0.001	3.29 (1.65–6.59)	0.001	2.74 (1.72–4.37)	< 0.001	2.30 (1.42–3.71)	0.001
*Chemotherapy*								
Yes vs. no	2.32 (1.61–3.34)	< 0.001	1.71 (1.14–2.57)	0.01	1.68 (1.21–2.33)	< 0.01	1.76 (1.21–2.57)	< 0.01
*APOBEC3B expression*								
High vs. low	2.72 (1.89–3.90)	< 0.001	1.93 (1.33–2.81)	0.001	1.89 (1.40–2.56)	< 0.001	1.45 (1.06–1.99)	0.02

Low APOBEC3B protein expression is defined as 0–10%; high protein expression is defined as > 10%

Furthermore, we performed a sensitivity analysis among the lymph node negative patients, as these patients were not treated with any systemic therapy. The univariable analysis showed that A3B-high tumors were associated with a shorter DFS (HR = 1.63; 95%CI = 1.16–2.27, log-rank test *p* = 0.004), MFS (HR = 2.17; 95%CI = 1.42–3.34, log-rank test *p* = 0.003), BCSS (HR = 3.19; 95%CI = 1.81–5.62, log-rank test *p* < 0.001) and OS (HR = 1.67; 95%CI = 1.06–2.61, log-rank test *p* = 0.024). In the multivariable analyses, only shorter MFS (HR = 1.57; 95%CI = 1.00–2.46) and BCSS (HR = 2.20; 95%CI = 1.18–4.10) remained significant (Supplementary Table 3–4). In addition, there was a borderline significant shorter DFS (HR = 1.36; 95%CI = 0.96–1.95) found in A3B-high samples compared to A3B-low samples.

When further stratifying the A3B expression including a group of samples with a protein expression over 25%, we found similar results for all patients and the lymph node negative patients (Supplementary Table 3–4). In both the univariable analyses and the multivariable analyses, there is a trend visible where samples with an increasing A3B protein expression are associated with worse outcomes compared to A3B-low samples. For example, the multivariable analysis showed a shorter BCSS for the A3B-I (HR = 1.62; 95%CI = 1.02–2.58) and A3B-H samples (HR = 2.24; 95%CI = 1.46–3.41) compared to the A3B-L samples (Supplementary Table 3).

### Analysis of A3B protein expression with PFS in patients receiving tamoxifen as first-line treatment

#### Patient characteristics

For this study, 220 samples from ER-positive BC patients were eligible, of whom 115 (52.3%) were A3B-low and 105 (47.7%) were A3B-high. 62 out of the 115 A3B-low samples had no A3B expression. Out of the A3B-high samples, 57 had expression in 11–25% of the cells, 32 in 26–50% of the cells and 16 had expression in more than 50% of the cells. The mean age at start of first-line tamoxifen treatment was lower in the A3B-high group (57.5 ± 11.9 years) compared to the A3B-low group (61.4 ± 13.0 years). The A3B-low group had a longer disease-free interval compared to the A3B-high group (45.2% of the A3B-low relapsed after 3 years compared to 24.8% within the A3B-high group). In this cohort, significant trends were found for age at start first-line tamoxifen and disease-free interval (Table 3). When further stratifying the samples with an A3B expression of more than 25% (Supplementary Table 5), we showed that there is a trend where higher A3B expression is associated with a shorter disease-free interval.

**Table 3:** Characteristics of the 220 ER-positive breast cancer patients treated with tamoxifen as part of the first-line treatment in the palliative setting included in the analysis and the test for trend between the characteristics and APOBEC3B protein expression

	Low (n = 115)	High (n = 105)	Test for trend *p*-value
Age at start first-line tamoxifen, in years			0.03
Median (range)	60 (28–87)	57 (26–78)	
Mean ± SD	61.4 ± 13.0	57.5 ± 11.9	
Age at start first-line tamoxifen, n (%)			0.12
≤ 55	44 (38.3)	45 (42.9)	
56–70	37 (32.2)	43 (41.0)	
> 70	34 (29.6)	17 (16.2)	
Menopausal status, n (%)			0.11
Premenopausal	24 (20.9)	32 (30.5)	
Postmenopausal	91 (79.1)	73 (69.5)	
Disease-free interval			< 0.01
< 1 year	19 (16.5)	22 (21.0)	
1–3 years	44 (38.3)	57 (54.3)	
> 3 years	52 (45.2)	26 (24.8)	
Dominant site of relapse, n (%)			0.09
Local regional	13 (11.3)	13 (12.4)	
Bone	67 (58.3)	45 (42.9)	
Other distant metastasis	35 (30.4)	47 (44.8)	

Low APOBEC3B protein expression is defined as 0–10%; high protein expression is defined as > 10%. Percentages may not add up to 100% due to rounding

## Association with PFS

**Fig. 3:**
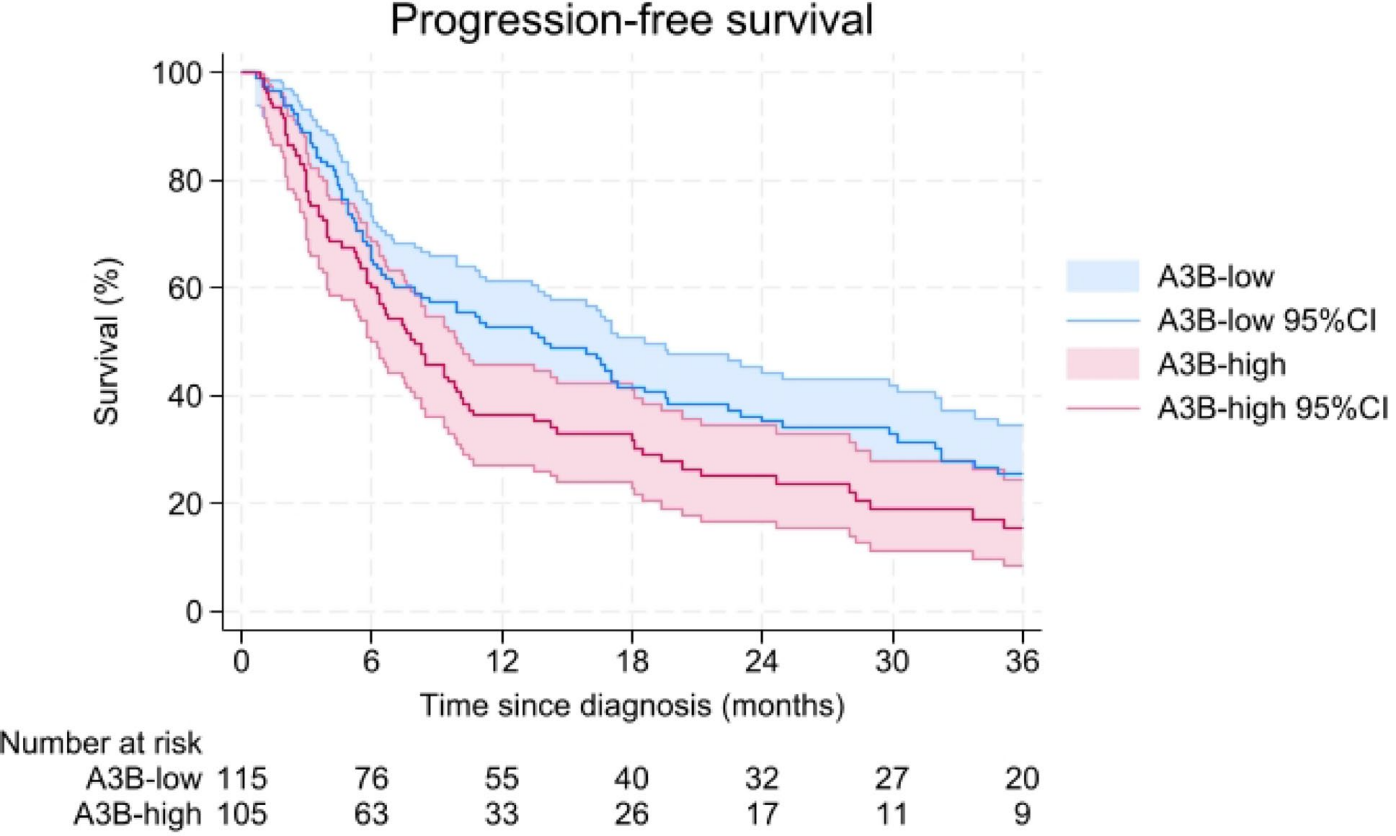
Progression-free survival of first-line Tamoxifen treatment, up to 36 months after the time of diagnosis of metastatic disease in primary ER-positive breast cancer patients. 95%CI = 95% confidence interval

In the univariate analysis, patients with A3B-high tumors were associated with a shorter PFS (HR = 1.41; 95%CI = 1.03–1.92, log-rank test *p* = 0.03, Fig. 3) compared to patients with A3B-low tumors. In the multivariable analysis adjusted for the age at start first-line tamoxifen, disease-free interval and dominant site of relapse, this effect did not remain significant (HR = 1.19; 95%CI = 0.86–1.65, Table 4).

**Table 4:** Univariable and multivariable analysis on progression-free survival in 220 ER-positive breast cancer patients who were treated with tamoxifen as part of the first-line treatment in the palliative setting

	Progression-free survival			
	Univariable analysis		Multivariable analysis	
	HR (95%CI)	*p*-value	HR (95%CI)	*p*-value
Age at start first-line tamoxifen, for each year increase	0.97 (0.96–0.98)	< 0.001	(0.96–0.98)	< 0.001
*Disease-free interval*				
< 1 year	Ref		Ref	
1–3 years	0.88 (0.58–1.33)	0.79	0.69 (0.45–1.06)	0.09
> 3 years	0.62 (0.39–0.96)	0.44	0.56 (0.35–0.89)	0.02
*Dominant site of relapse*				
Local regional	Ref		Ref	
Bone	2.64 (1.37–5.08)	< 0.01	2.56 (1.33–4.94)	0.01
Other distant metastasis	2.48 (1.27–4.85)	< 0.01	2.56 (1.29–5.09)	0.01
*APOBEC3B expression*				
High vs. low	1.41 (1.03–1.92)	0.03	1.19 (0.86–1.65)	0.30

Low APOBEC3B protein expression is defined as 0–10%; high protein expression is defined as > 10%

By further stratifying the A3B expression, we found a trend between higher A3B expression and a shorter PFS (HR = 1.37; 95%CI = 0.95–1.97 for A3B-I and HR = 1.46; 95%CI = 0.98–2.18 for A3B-H compared toA3B-L, log-rank test p = 0.09). After adjustment for potential confounders, a similar trend was found (HR = 1.13; 95%CI = 0.77–1.66 for A3B-I and HR = 1.28; 95%CI = 0.84–1.93 for A3B-H compared to A3B-L, Supplemental Table 6).

## Discussion

In two historical cohorts of ER-positive BC patients, we studied the impact of A3B expression on (1) the prognostic value in non-metastatic primary BC patients who did not receive any systemic endocrine therapy as part of their standard of care treatment and (2) PFS after first-line palliative treatment with tamoxifen. The quantity of A3B expression on TMAs was used to categorize samples into those having high A3B expression vs those having low A3B expression. In the prognostic cohort, we found that BC patients with A3B-high protein expression were more often diagnosed at a higher stage of disease and higher mitotic activity. Furthermore, we found that increased A3B expression at the protein level is also associated with a worse prognosis. This was also found in the lymph node negative patients who at that time did not receive any adjuvant systemic therapy, allowing us to study the pure prognostic value of A3B expression. Furthermore, in the second cohort, we found that an increased A3B expression was associated with a shorter PFS. This underscores the prognostic value of A3B protein expression using the monoclonal antibody 5210–87-13 [23].

Also, we found a significant trend between A3B protein expression and tumor size, tumor grade and mitotic rate. These findings are in line with previous findings that A3B is linked to cellular proliferation [16, 20, 26], regulated by E2F transcription factors, and on a cellular level, co-expressed in the nucleus with proliferation markers such as Ki67 and cyclin B1 [16, 27–29].

In the main analyses, we dichotomized A3B protein expression arbitrarily by the cut-off of 10% for positivity. In the supplementary analyses, we also studied the group who had a A3B positive expression of more than 25%, showing a clear trend of increasing A3B expression and worse outcome. After adjustment for age at diagnosis, menopausal status, tumor size, tumor grade and treatment with chemotherapy, this trend remained visible, however, part of the risk estimates became non-significant. This could be caused by the cut-off used in the analysis. As this is the first study to examine the impact of A3B expression on prognosis and PFS, there might a more optimal cut-off. Especially the A3B-I group (11–25% expression) might be a heterogeneous group, and therefore difficult to properly stratify them. Furthermore, the lack of significant effects could also be caused by the small sample size (e.g. 48 out of the 220 samples were A3B-H in the PFS analysis). Further research in a larger population is needed to determine the best cut-off for A3B expression to study the prognosis and PFS.

Overall, our results are in line with our earlier findings showing that elevated A3B mRNA expression levels are associated with a worse prognosis [18, 21]. However, the majority of the previous studies are on the prognostic value of A3B studied by the mRNA expression [17–19, 30], rather than protein expression. One study combined the protein expression with other data, including mRNA expression and the presence of tumor-infiltrating lymphocytes [17]. They examined A3B mRNA and protein expression in 116 unselected BC samples. Results showed that the protein and mRNA levels for A3B were similar, suggesting that prognostic value found using mRNA could be extrapolated to protein expression. However, they did not study the association between A3B protein expression and prognosis.

This is the first study to determine A3B protein expression on FFPE tissues and its association in uni- and multivariable analysis with prognosis and with progression during endocrine treatment in ER-positive BC patients. Also, by using a historical cohort in BC patients who did not receive any systemic (neo-)adjuvant treatment, we had a unique opportunity to study the pure prognostic value of A3B protein. As a result, these results are not directly generalizable to current patients as we were not able to take all the current treatment regimens into account. However, based on recent studies [14–21], we would still expect a worse prognosis and shorter PFS in ER-positive BC patients with high levels of A3B expression.

To conclude, A3B protein expression is associated with a poor outcome, disease progression and rapid progression on first-line tamoxifen treatment in ER-positive BC patients. This underlines the importance of the A3B protein, a source of APOBEC mutagenesis, as a prognostic factor in ER-positive BC patients and a marker of rapid progression on endocrine treatment in recurrent BC. The fact that A3B protein can be routinely measured using standard diagnostic IHC practice makes it a great candidate as a clinically useful biomarker.

## Supplementary Information

Additional file 1.

## Declarations

### Data availability

Data available on reasonable request.
