# Supplementary material for: APOBEC3B protein expression associates with poor prognosis for breast cancer patients with ER-positive disease
Source: Breast Cancer Res. 2025 Dec 9;28:7. doi: 10.1186/s13058-025-02167-7 (PMC12802011; doi:10.1186/s13058-025-02167-7)
Supplement: Supplementary file 1 — Additional file 1. [file 13058_2025_2167_MOESM1_ESM.docx]

**Supplementary Table 1**: Characteristics of the 646 samples included in the analysis, stratified by three categories of APOBEC3B protein expression (0-10%, 11-25% or >25% expression), and the test for trend between the characteristics and APOBEC3B protein expression.

|  | **APOBEC3B expression classification** | | |  |
| --- | --- | --- | --- | --- |
|  | Low  0-10%  (n=396) | Intermediate  11-25%  (n=125) | High  >25%  (n=125) | **Test for trend**  **P-value** |
| Age  Median (range)  Mean ± SD | 55 (27-92)  55.8 ± 12.4 | 57 (27-86)  56.8 ± 13.1 | 52 (26-84)  53.7 ± 12.1 | 0.29 |
| Age category, n (%)  ≤40  41-55  >55 | 39 (9.9)  165 (41.7)  192 (48.5) | 11 (8.8)  48 (38.4)  66 (52.8) | 15 (12.0)  61 (48.8)  49 (39.2) | 0.28 |
| Menopausal status, n (%)  Premenopausal  Postmenopausal | 217 (54.8)  179 (45.2) | 75 (60.0)  50 (40.0) | 65 (52.0)  60 (48.0) | 0.99 |
| Tumor size, n (%)  pT1  pT2/pT3 | 279 (70.5)  117 (29.6) | 70 (56.0)  55 (44.0) | 54 (43.2)  71 (56.8) | <0.001 |
| Tumor grade, n (%)  1  2/3 | 129 (32.6)  267 (67.4) | 15 (12.0)  110 (88.0) | 10 (8.0)  115 (92.0) | <0.001 |
| Chemotherapy, n (%)  No or not applicable  Yes | 317 (80.1)  79 (20.0) | 99 (79.2)  26 (20.8) | 91 (72.8)  34 (27.2) | 0.14 |
| Mitotic activity per mm^2^  Median (range)  Mean ± SD | 3 (0-70)  5.1 ± 7.4 | 6 (0-90)  8.3 ± 10.6 | 12 (0-80)  15.4 ± 14.3 | <0.001 |
| Mitotic activity per mm^2^, n (%)  0-10  >10 | 345 (87.6)  49 (12.4) | 93 (74.4)  32 (25.6) | 56 (44.8)  69 (55.2) | <0.001 |

APOBEC3B protein expression is categorized as 0-10%; 11-25% and >25%

**Supplementary Table 2:** Univariable and multivariable analysis for disease-free survival, metastatic-free survival, breast cancer-specific survival and overall survival in 384 ER-positive, lymph node negative breast cancer patients.

|  | **Disease-free survival** | | | | | **Metastatic disease-free survival** | | | |
| --- | --- | --- | --- | --- | --- | --- | --- | --- | --- |
|  | Univariable model | | Multivariable model | | Univariable model | | | Multivariable model | |
|  | HR (95%CI) | P-value | HR (95%CI) | P-value | HR (95%CI) | | P-value | HR (95%CI) | P-value |
| Age, each year increase | 0.99 (0.98-1.01) | 0.27 | 0.98 (0.96-1.01) | 0.24 | 0.99 (0.97-1.01) | | 0.21 | 0.97 (0.94-1.00) | 0.10 |
| Menopausal status  Post- vs premenopausal | 0.95 (0.68-1.33) | 0.77 | 1.28 (0.71-2.33) | 0.41 | 0.97 (0.63-1.49) | | 0.89 | 1.67 (0.77-3.63) | 0.19 |
| Tumor size  pT2/pT3 vs. pT1 | 1.49 (1.04-2.11) | 0.03 | 1.35 (0.94-1.94) | 0.11 | 2.08 (1.35-3.20) | | 0.001 | 1.77 (1.13-2.76) | 0.01 |
| Tumor grade (BR)  2/3 vs. 1 | 1.80 (1.18-2.73) | <0.01 | 1.55 (1.00-2.39) | 0.05 | 3.51 (1.76-7.02) | | <0.001 | 2.72 (1.34-5.53) | <0.01 |
| APOBEC3B expression  High vs. low | 1.63 (1.16-2.27) | <0.01 | 1.36 (0.96-1.95) | 0.09 | 2.17 (1.42-3.34) | | <0.001 | 1.57 (1.00-2.46) | 0.05 |
|  | **Breast cancer-specific survival** | | | | **Overall survival** | | | | |
|  | Univariable model | | Multivariable model | | Univariable model | | | Multivariable model | |
|  | HR (95%CI) | P-value | HR (95%CI) | P-value | HR (95%CI) | | P-value | HR (95%CI) | P-value |
| Age, each year increase | 0.98 (0.96-1.00) | 0.12 | 0.98 (0.94-1.03) | 0.42 | 1.02 (1.00-1.04) | | 0.08 | 1.02 (0.99-1.05) | 0.26 |
| Menopausal status  Post- vs premenopausal | 0.76 (0.44-1.31) | 0.32 | 1.11 (0.40-3.10) | 0.84 | 1.37 (0.86-2.19) | | 0.18 | 0.98 (0.43-2.21) | 0.95 |
| Tumor size  pT2/pT3 vs. pT1 | 2.36 (1.36-4.09) | <0.01 | 1.76 (1.00-3.10) | 0.05 | 1.99 (1.27-3.13) | | <0.01 | 1.69 (1.06-2.70) | 0.03 |
| Tumor grade (BR)  2/3 vs. 1 | 10.16 (2.47-41.79) | 0.001 | 7.04 (1.69-29.40) | <0.01 | 2.49 (1.31-4.71) | | <0.01 | 2.22 (1.15-4.29) | 0.02 |
| APOBEC3B expression  High vs. low | 3.19 (1.81-5.62) | <0.001 | 2.18 (1.21-3.91) | <0.01 | 1.67 (1.06-2.61) | | 0.03 | 1.32 (0.83-2.10) | 0.25 |

Low APOBEC3B protein expression is defined as 0-10%; high protein expression is defined as >10%.

**Supplemental Table 3:** Univariable and multivariable analysis for disease-free survival, metastatic-free survival, breast cancer-specific survival and overall survival in 645 ER-positive breast cancer patients

|  | Disease-free survival | | | | | | Metastatic disease-free survival | | | |
| --- | --- | --- | --- | --- | --- | --- | --- | --- | --- | --- |
|  | Univariable analysis | | | Multivariable analysis | | | Univariable analysis | | Multivariable analysis | |
|  | HR (95%CI) | P-value | HR (95%CI) | | P-value | HR (95%CI) | | P-value | HR (95%CI) | P-value |
| Age, each year increase | 0.99 (0.98-1.00) | <0.01 | 0.98 (0.96-1.00) | | 0.03 | 0.98 (0.97-1.00) | | 0.01 | 0.98 (0.96-1.00) | 0.10 |
| Menopausal status  Post- vs premenopausal | 0.83 (0.65-1.06) | 0.14 | 1.22 (0.81-1.83) | | 0.35 | 0.86 (0.64-1.14) | | 0.29 | 1.37 (0.85-2.22) | 0.20 |
| Tumor size  pT2/pT3 vs. pT1 | 1.71 (1.34-2.18) | <0.001 | 1.48 (1.15-1.91) | | <0.01 | 2.20 (1.66-2.93) | | <0.001 | 1.78 (1.32-2.40) | <0.001 |
| Tumor grade (BR)  2/3 vs. 1 | 1.99 (1.43-2.76) | <0.001 | 1.68 (1.20-2.35) | | <0.01 | 3.03 (1.92-4.77) | | <0.001 | 2.40 (1.51-3.73) | <0.001 |
| Chemotherapy | 1.64 (1.26-2.14) | <0.001 | 1.41 (1.04-1.90) | | 0.03 | 2.00 (1.48-2.71) | | <0.001 | 1.70 (1.21-2.40) | <0.01 |
| APOBEC3B expression  Low  Intermediate  High | Ref.  1.60 (1.18-2.16)  1.76 (1.30-2.37) | <0.01  <0.001 | Ref.  1.38 (1.01-1.87)  1.36 (0.99-1.86) | | 0.04  0.06 | Ref.  1.80 (1.27-2.56)  2.06 (1.46-2.92) | | 0.001  <0.001 | Ref.  1.42 (0.99-2.03)  1.42 (0.99-2.04) | 0.06  0.06 |
|  | Breast cancer-specific survival | | | | | | Overall survival | | | |
|  | Univariable analysis | | | Multivariable analysis | | | Univariable analysis | | Multivariable analysis | |
|  | HR (95%CI) | P-value | HR (95%CI) | | P-value | HR (95%CI) | | P-value | HR (95%CI) | P-value |
| Age, each year increase | 0.98 (0.96-0.99) | <0.01 | 0.99 (0.97-1.02) | | 0.59 | 1.01 (0.99-1.02) | | 0.97 | 1.02 (1.00-1.05) | 0.04 |
| Menopausal status  Post- vs premenopausal | 0.67 (0.47-0.96) | 0.03 | 0.91 (0.50-1.67) | | 0.77 | 1.10 (0.81-1.49) | | 0.54 | 0.86 (0.51-1.45) | 0.58 |
| Tumor size  pT2/pT3 vs. pT1 | 2.98 (2.07-4.28) | <0.001 | 2.14 (1.47-3.11) | | <0.001 | 2.36 (1.74-3.20) | | <0.001 | 1.83 (1.33-2.51) | <0.001 |
| Tumor grade (BR)  2/3 vs. 1 | 4.69 (2.38-9.24) | <0.001 | 3.28 (1.64-6.56) | | 0.001 | 2.74 (1.72-4.37) | | <0.001 | 2.28 (1.41-3.69) | 0.001 |
| Chemotherapy | 2.32 (1.61-3.34) | <0.001 | 1.71 (1.14-2.57) | | 0.01 | 1.68 (1.21-2.33) | | <0.01 | 1.77 (1.21-2.59) | <0.01 |
| APOBEC3B expression  Low  Intermediate  High | Ref.  2.11 (1.34-3.33)  3.38 (2.25-5.08) | 0.001  <0.001 | Ref.  1.62 (1.02-2.58)  2.24 (1.46-3.41) | | 0.04  <0.001 | Ref.  1.47 (0.99-2.19)  2.34 (1.65-3.32) | | 0.06  <0.001 | Ref.  1.18 (0.79-1.76)  1.75 (1.22-2.53) | 0.43  <0.01 |

APOBEC3B protein expression is categorized as Low (0-10%); Intermediate (11-25%) and High (>25%)

**Supplementary Table 4:** Univariable and multivariable analysis for disease-free survival, metastatic-free survival, breast cancer-specific survival and overall survival in 384 ER-positive, lymph node negative breast cancer patients.

|  | **Disease-free survival** | | | | | **Metastatic disease-free survival** | | | |
| --- | --- | --- | --- | --- | --- | --- | --- | --- | --- |
|  | Univariable model | | Multivariable model | | Univariable model | | | Multivariable model | |
|  | HR (95%CI) | P-value | HR (95%CI) | P-value | HR (95%CI) | | P-value | HR (95%CI) | P-value |
| Age, each year increase | 0.99 (0.98-1.01) | 0.27 | 0.99 (0.96-1.01) | 0.24 | 0.99 (0.97-1.01) | | 0.21 | 0.97 (0.94-1.01) | 0.10 |
| Menopausal status  Post- vs premenopausal | 0.95 (0.68-1.33) | 0.77 | 1.28 (0.71-2.33) | 0.41 | 0.97 (0.63-1.49) | | 0.89 | 1.66 (0.76-3.61) | 0.20 |
| Tumor size  pT2/pT3 vs. pT1 | 1.49 (1.05-2.11) | 0.03 | 1.34 (0.93-1.93) | 0.11 | 2.08 (1.35-3.20) | | 0.001 | 1.74 (1.11-2.73) | 0.02 |
| Tumor grade (BR)  2/3 vs. 1 | 1.80 (1.18-2.73) | <0.01 | 1.55 (1.00-2.39) | 0.05 | 3.51 (1.76-7.02) | | <0.001 | 2.75 (1.35-5.58) | <0.01 |
| APOBEC3B expression  Low  Intermediate  High | Ref.  1.54 (1.02-2.31)  1.74 (1.14-2.67) | 0.04  0.01 | Ref.  1.31 (0.86-2.00)  1.44 (0.92-2.24) | 0.21  0.11 | Ref.  1.92 (1.14-3.23)  2.51 (1.49-4.22) | | 0.01  0.001 | Ref.  1.43 (0.84-2.44)  1.75 (1.02-3.01) | 0.19  0.04 |
|  | **Breast cancer-specific survival** | | | | **Overall survival** | | | | |
|  | Univariable model | | Multivariable model | | Univariable model | | | Multivariable model | |
|  | HR (95%CI) | P-value | HR (95%CI) | P-value | HR (95%CI) | | P-value | HR (95%CI) | P-value |
| Age, each year increase | 0.98 (0.96-1.00) | 0.12 | 0.99 (0.94-1.03) | 0.47 | 1.02 (1.00-1.04) | | 0.08 | 1.02 (0.99-1.06) | 0.21 |
| Menopausal status  Post- vs premenopausal | 0.76 (0.44-1.31) | 0.32 | 1.09 (0.39-3.06) | 0.88 | 1.37 (0.86-2.19) | | 0.18 | 0.94 (0.41-2.14) | 0.88 |
| Tumor size  pT2/pT3 vs. pT1 | 2.36 (1.36-4.09) | <0.01 | 1.70 (0.96-3.00) | 0.07 | 1.99 (1.27-3.13) | | <0.01 | 1.63 (1.02-2.61) | 0.04 |
| Tumor grade (BR)  2/3 vs. 1 | 10.16 (2.47-41.79) | 0.001 | 7.12 (1.72-29.96) | <0.01 | 2.49 (1.31-4.71) | | <0.01 | 2.25 (1.16-4.34) | 0.02 |
| APOBEC3B expression  Low  Intermediate  High | Ref.  2.47 (1.24-4.93)  4.12 (2.16-7.84) | 0.01  <0.001 | Ref.  1.75 (0.87-3.53)  2.73 (1.40-5.35) | 0.12  <0.01 | Ref.  1.31 (0.74-2.34)  2.12 (1.24-3.62) | | 0.36  <0.01 | Ref.  1.04 (0.58-1.88)  1.69 (0.97-2.96) | 0.89  0.07 |

APOBEC3B protein expression is categorized as Low (0-10%); Intermediate (11-25%) and High (>25%)

**Supplementary Table 5:** Characteristics of the 220 ER-positive breast cancer patients treated with tamoxifen as part of the first-line treatment in the palliative setting included in the analysis and the test for trend between the characteristics and APOBEC3B protein expression

|  | **APOBEC3B expression classification** | | |  |
| --- | --- | --- | --- | --- |
|  | Low  0-10%  (n=115) | Intermediate  11-25%  (n=57) | High  >25%  (n=48) | **Test for trend**  **p-value** |
| Age at start first-line tamoxifen, in years  Median (range)  Mean ± SD | 60 (28-87)  61.4 ± 13.0 | 56 (34-80)  56.9 ± 11.8 | 57 (37-81)  58.3 ± 12.0 | 0.24  0.29 |
| Age at start first-line tamoxifen, n (%)  ≤55  56-70  >70 | 44 (38.3)  37 (32.2)  34 (29.6) | 27 (47.4)  21 (36.8)  9 (15.8) | 18 (37.5)  22 (45.8)  8 (16.7) | 0.14 |
| Menopausal status, n (%)  Premenopausal  Postmenopausal | 24 (20.9)  91 (79.1) | 18 (31.6)  39 (68.4) | 14 (29.2)  34 (70.8) | 0.25 |
| Disease-free interval  <1 year  1-3 years  >3 years | 19 (16.5)  44 (38.3)  52 (45.2) | 9 (15.8)  31 (54.4)  17 (29.8) | 13 (27.1)  26 (54.2)  9 (18.8) | 0.01 |
| Dominant site of relapse, n (%)  Local regional  Bone  Other distant metastasis | 13 (11.3)  67 (58.3)  35 (30.4) | 5 (8.8)  23 (40.4)  29 (50.9) | 8 (16.7)  22 (45.8)  18 (37.5) | 0.08 |

APOBEC3B protein expression is categorized as Low (0-10%); Intermediate (11-25%) and High (>25%)

**Supplementary Table 6:** Univariable and multivariable analysis on progression-free survival in 220 ER-positive breast cancer patients who were treated with tamoxifen as part of the first-line treatment in the palliative setting.

|  | **Progression-free survival** | | | |
| --- | --- | --- | --- | --- |
|  | Univariable analysis | | Multivariable analysis | |
|  | HR (95%CI) | p-value | HR (95%CI) | p-value |
| Age at start first-line tamoxifen, for each year increase | 0.97 (0.96-0.98) | <0.001 | 0.97 (0.96-0.98) | <0.001 |
| Disease-free interval  <1 year  1-3 years  >3 years | Ref.  0.88 (0.58-1.33)  0.62 (0.39-0.96) | 0.79  0.44 | Ref.  0.69 (0.45-1.06)  0.57 (0.36-0.90) | 0.09  0.02 |
| Dominant site of relapse  Local regional  Bone  Other distant metastasis | Ref.  2.64 (1.37-5.08)  2.48 (1.27-4.85) | <0.01  <0.01 | Ref.  2.56 (1.33-4.94)  2.57 (1.30-5.10) | 0.01  0.01 |
| APOBEC3B expression  Low  Intermediate  High | Ref.  1.37 (0.95-1.97)  1.46 (0.98-2.18) | 0.10  0.06 | Ref.  1.13 (0.77-1.66)  1.28 (0.84-1.93) | 0.52  0.25 |

APOBEC3B protein expression is categorized as Low (0-10%); Intermediate (11-25%) and High (>25%)
